# How collective reward structure impedes group decision making: An experimental study using the HoneyComb paradigm

**DOI:** 10.1371/journal.pone.0259963

**Published:** 2021-11-16

**Authors:** Marie Ritter, Meng Wang, Johannes Pritz, Olaf Menssen, Margarete Boos

**Affiliations:** Social and Communication Psychology, Georg-Elias-Müller-Institute for Psychology, Georg-August-University Göttingen, Göttingen, Lower Saxony, Germany; Teesside University, UNITED KINGDOM

## Abstract

This study investigates if and under which conditions humans are able to identify and follow the most advantageous leader who will them provide with the most resources. In an iterated economic game with the aim of earning monetary reward, 150 participants were asked to repeatedly choose one out of four leaders. Unbeknownst to participants, the leaders were computer-controlled and programmed to yield different expected payout values that participants had to infer from repeated interaction over 30 rounds. Additionally, participants were randomly assigned to one of three conditions: single, independent, or cohesion. The conditions were designed to investigate the ideal circumstances that lead to identifying the most advantageous leader: when participants are alone (single condition), in a group that lets individuals sample information about leaders independently (independent condition), or in a group that is rewarded for cohesive behavior (cohesion condition). Our results show that participants are generally able to identify the most advantageous leader. However, participants who were incentivized to act cohesively in a group were more likely to settle on a less advantageous leader. This suggests that cohesion might have a detrimental effect on group decision making. To test the validity of this finding, we explore possible explanations for this pattern, such as the length of exploration and exploitation phases, and present techniques to check for confounding factors in group experiments in order to identify or exclude them as alternative explanations. Finally, we show that the chosen reward structure of the game strongly affects the observed following behavior in the group and possibly occludes other effects. We conclude with a recommendation to carefully choose reward structures and evaluate possible alternative explanations in experimental group research that should further pursue the study of exploration/exploitation phases and the influence of group cohesion on group decision making as promising topics for further research.

## Introduction

Democracies rely on the basic idea that elections will lead to the establishment of a government most likely to make the best decisions on behalf of its voters. Voters supposedly choose their representatives on an estimate of how well they will provide themselves and their demographic group with benefits [1] based on public information and their previous experience with the party [2]. Thus, the election is presumed to manifest a pooled "wisdom of the crowd" judgment that surpasses individual judgments and thereby contributes to the entire group’s welfare [3]. However, history and recent examples have demonstrated that election choices are observably more complex and often fail to produce leaders that are beneficial to their followers [4].

In this study, we investigate how human groups coordinate when faced with a number of differently advantageous leaders and need to infer each leader’s qualities from their continuously observed behavior. Specifically, we examine the participants’ ability to identify the leader that will see them to their best possible result under three different conditions.

### Leadership as a result of group decision making

Leadership can be construed as the result of choices followers make in consideration of their objective to maximize their own and their group’s advantage. Leaders and followers emerge in everyday decision making as an adaptive solution that enables groups to decisively initiate, arrive at, and complete the best possible collective action goals [5]. In other words, leadership and followership occur as they are part of the same interdependent agreement that groups form to coordinate significant collective actions that provide all group members with benefits upon completion [6,7]. Previous studies have shown that leadership can emerge in accordance with followers’ needs [8,9].

An extensive body of literature in evolutionary psychology has proposed that leader-follower interactions arise naturally in (leaderless) animal groups with coordination needs such as joint migration or foraging [10,11]. For humans, evolutionary adaptation took place in small, semi-nomadic family groups [12]. In these environments, leading and following behaviors provided humans as a group living species with fitness advantages such as higher group effectiveness [12–20]. As there was no formalized leadership, leaders gained their influence on collective action by demonstrating their expertise [21–25]. Thus, evolutionary research would suggest the existence of an evolved psychological mechanism that enables followers to assess different potential leaders and to select the most appropriate individual to follow [26].

Applied to human behavior today, these findings indicate that humans can use heuristics and decision rules in choosing their leaders which can increase the efficiency of leaders and followers in their respective roles [15,18]. Using these psychological mechanisms, humans are able to reap the benefits of coordinated group actions while at the same time mitigating its costs, such as coordination efforts.

### Finding the best leader

When presented with a number of options and no information on which option is best, individuals still arrive at economically sound decisions by adjusting their behavior to maximize the overall or average benefit a choice offers [27–30].

Previous work has shown that other people’s behavioral tendencies (i.e., typical behaviors) are stored and represented on a neural basis [31,32]. This is especially true in iterative economic games, where the profit outcome of the entire game is dependent on both one’s own recurrent action and how other parties respond [33]. In such games, a player’s ability to anticipate the behavior of other players often correlates with their potential to maximize personal profit [34]. Existing research has shown that predictions of someone else’s behavioral tendencies rely on learning from this person’s past behavior [35]. Several studies suggest that with repeated exposure to another’s behavior, participants are able to infer how others are likely to behave in a given context [36]; participants are more likely to continue associating with those individuals with whom previous interactions yielded a positive outcome [37–39].

Similar processes may be at play when followers are predicting prospective contributions of specific leaders to the achievement of collective group goals from a leader’s past behavior. In repeated encounters with distinct leaders, group members may develop an internal model of expected value attainable by aligning their own action to the leader’s behavior. This internal model may facilitate a comparative process, allowing the individual to adaptively identify the leader with the highest potential for contributing towards a given task.

### … in moving human groups

The aforementioned “wisdom of the crowd” might also play a role in movement behavior. For example, group navigation performance is improved through “the pooling of information from many inaccurate compasses” [40] and group cohesion can suppress navigation errors in groups that allow individuals to make independent suggestions on which direction to take [41]. This “many wrongs principle” of navigation has been confirmed empirically in groups of both birds and humans [42,43]. Moussaid and colleagues show that self-organized collective behavior rely on local interactions between individuals who then integrate this information on the collective level [44]. It seems that interactions on a movement-only basis result in an averaging process that combines individual information to arrive at the optimal choice.

For human groups, using a multi-client simulated environment of a visual field, Boos and colleagues demonstrated that followers are indeed able to identify advantageous leaders by their movement patterns and, thereby reach an optimal goal [45]. A minority of informed group members who were given information about the location of a more profitable reward field were able to make an uninformed majority follow by displaying certain movement patterns (moving first and moving cohesively). This finding is in line with previous results using face-to-face groups [46,47].

These simple decision rules (e.g., the first-mover effect) are crucial in explaining how collective movement decisions are made. Additionally, some studies illuminate how people move when a group incorporates several potential leaders [46,48]. Consensus exists in literature that movement direction of the group reflects an aggregate decision-making process where each individual balances their indented movement direction with the movement direction of other group members. Moreover, group movement is influenced by the quorum response [49]: Group movement decisions toward one direction are adhered to by subsequent decision makers once a certain threshold of decision makers has been met. Ultimately, decisions of individuals incorporate their responses to both the environment and other individuals’ movement choices [44,48]. This means that individuals may need to find an appropriate balance between acquiring private information about prospective leaders through exploration and relying on social information from observing others.

### Current study

In the current study, we adapted the HoneyComb paradigm by Boos and colleagues [45,50] to serve as our investigative platform. The HoneyComb paradigm is a multi-agent computer-based virtual game platform that was designed to eliminate all sensory and communication channels except the perception of participant-assigned avatar movements on the playfield. We decided to use the HoneyComb paradigm in this study as we assume that it is a highly suitable tool to research the process of group decision making. To do that, the HoneyComb paradigm records spatio-temporal data to track the movement of members of a real group. In fact, we believe that only few other tools are suitable to investigate the process of group decision making, such as group interaction analysis [51]. However, this requires time-intensive analysis and introduces confounds into communication between group members that the restricted experimental environment of HoneyComb can control.

In the adapted version of the HoneyComb paradigm, participants were asked to move on a virtual playing field where they had to repeatedly choose between following four distinct leaders. These leaders appeared to know the location of four reward fields that participants themselves could not see. Unbeknownst to the participants, the four leaders were computer-controlled and had different predefined chances of arriving at a profitable reward field. Consequently, participants could maximize their profit by learning about the leaders’ overall decision quality. Using this paradigm, we aim to test if and under what conditions followers can learn from repeatedly experiencing the consequences of their following behavior. In particular, we aim to investigate if followers can learn to follow those leaders more frequently whose decisions result in the followers’ greatest overall payoff.

We expect that participants will be able to identify the most advantageous leader. Specifically, we hypothesize that participants will more frequently follow the computer-controlled leader who is programmed to yield the highest expected reward for their followers. Note that we explicitly define following as a behavior (i.e., an individual gives his/her support to an initiator for a certain activity) and not as a motivation or preference [52].

To further explore participants’ behavior in different contexts, we created three conditions: In the first condition, participants played the game by themselves (*single condition*). The pre-programmed leaders were the only others that were visible in this condition. In the second condition, participants played the iterative game in the presence of five other participants (*independent condition*). In the third condition, participants also played the game in the presence of five other participants but were rewarded on a group level when they moved cohesively towards a reward field (*cohesion condition*). With this design, we aim to find optimal conditions for participants to identify the best leader; either by themselves (single condition), in a group that favors private experience over social information from the group in the pooling of information (independent condition), or a condition that emphasizes cohesion early on (cohesion condition) and may in this way reduce the individuals’ propensity to gather private information.

## Methods

### Sample

Overall, we recruited 156 participants (51.3% female) in groups of twelve. Six participants had to be excluded because they left before completion of the experiment. The remaining sample consisted of 150 participants (*M* = 21.90, *SD* = 3.05), of which 50.7% were female. Participants were compensated according to the amount of virtual money they earned through their in-game behavior. All participants were informed about the procedure of the study and gave written consent to participate. Data collection and data analysis procedures in this study were approved by the Ethics Committee of the Georg-Elias-Müller Institute for Psychology of the University of Göttingen (proposal 10/2016).

### Procedure and experimental setup

**Fig 1:**
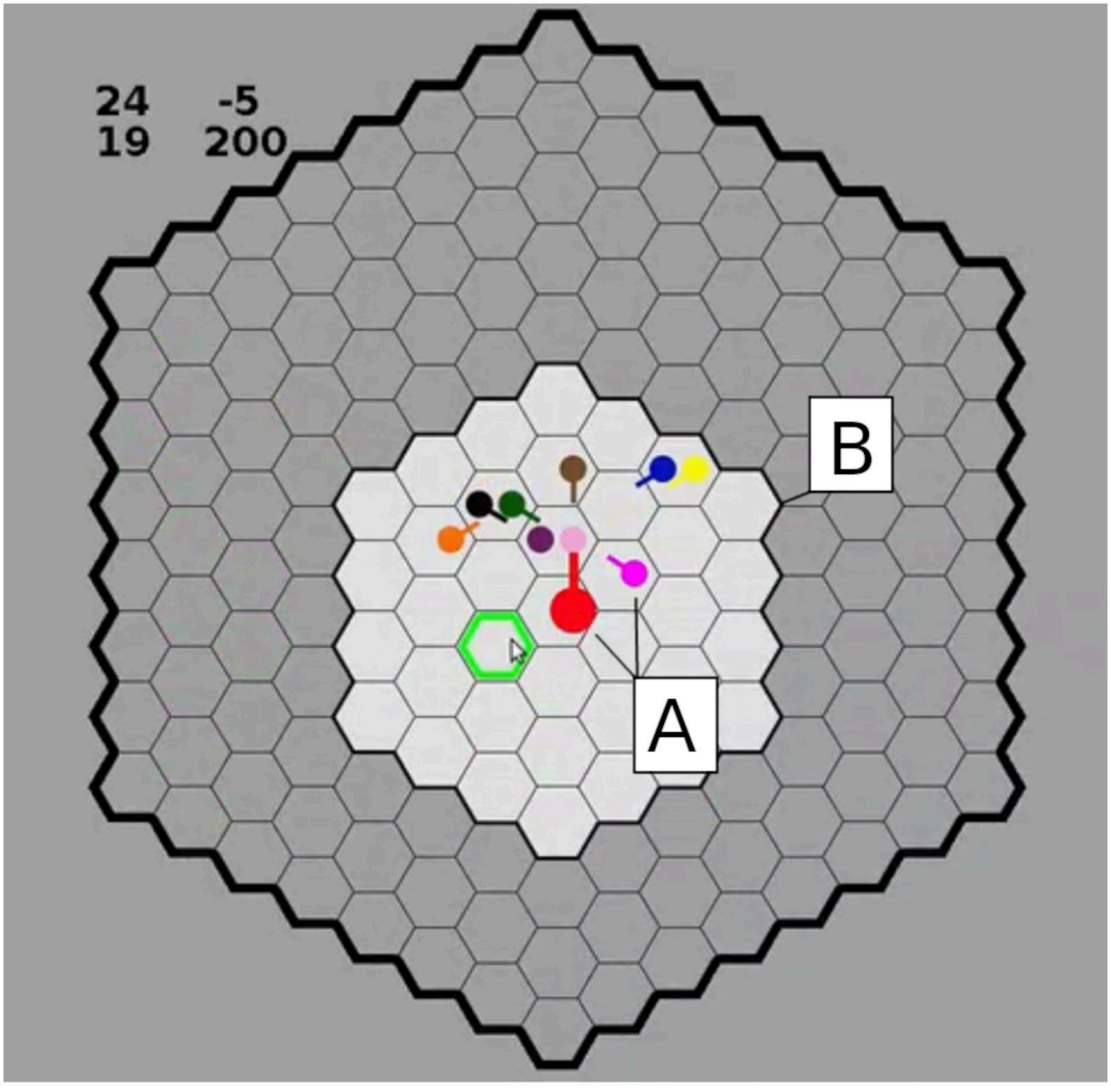
The HoneyComb virtual playing field. A–Each player controlled a differently-colored avatar. B–A visual radius of two fields surrounded each player.

The HoneyComb virtual experiment paradigm [45,50] was adapted to the purpose of the current study in the following way: This Iterated HoneyComb Game consisted of 30 consecutive rounds of the same virtual computer game. The game was played on a virtual field in the form of a honeycomb. On this virtual playing field, participants controlled avatars in the form of differently colored dots via the movement of their mouse. Communication among players was restricted to the visual perception of each other’s movements within a visual radius of two adjoining spatial fields surrounding their own avatar. The rules of the virtual game remained the same throughout the 30 rounds. Players were initially endowed with a small amount of money and were then instructed to maximize their payoff by arriving at fields that yielded a monetary reward at the end of each round. In order to counterbalance income effects, half of the participants were initially endowed with 50 cents, while the other half received 250 cents. A screenshot showing the virtual playing field can be seen in Fig 1; a detailed description of the experimental setup and procedure can be found in the S1 and S2 Texts, while an interactive payout matrix can be accessed in the S2 Table.

Participants were led to believe that four people in their group were chosen to receive additional information. These informed players were supposedly able to see fields with monetary rewards beyond other players’ visual radius and were allowed to move first. The instructions delineated that the four informed players were not allowed to aim toward the same reward field.

In reality, these four players were potential leaders with pre-programmed strategies of reward attainment of their followers with different expected values over the entire 30 rounds (EV, i.e., the average payout this leader would yield): The “incompetent” leader paid the participants 20 cents 20% of the time (EV = 120 cents). The “risky neutral” leader paid the participants 20 cents 45% of the time (EV = 270 cents), while the “secure neutral” leader paid 10 cents 90% of the time (EV = 270 cents). Lastly, the “competent” leader paid 20 cents 80% of the time (EV = 480 cents).

The participants were told that they could find the reward fields by following one of the four informed players. At the end of every round, players received feedback on their gains, losses, and the total amount of money on their account.

In order to explore the conditions under which participants are most likely to identify the competent leader, we designed three between-subject conditions (single, independent, and cohesion condition): In the single condition, participants played the game with only the four leaders present, while in the independent and cohesion condition, participants played the game with the four leaders and five other players.

In the independent condition, the rewards participants attained by arriving at a reward field were simply added to their in-game account. No further instructions or incentives to behave in a certain way were given. In contrast, participants in the cohesion condition received further reward for arriving on a reward field with other players. This additional reward was computed by multiplying the individual reward gained on the field by the number of players who arrived there. For instance, when four players arrived at the same reward field paying out 10 cents, each player received 10 cents x 4 players = 40 cents.

A post-game questionnaire assessed risk propensity [53], self-esteem [54], embodiment [55], and additional demographic questions (see S3 Text). As the results from the questionnaires are not relevant to the argument made in this report, they will not be reported here.

### Data analysis

Both, initial data processing and data analyses, were conducted using the statistics program R (R Core Team, 2018). A complete list of the used R packages can be found in the S4 Text.

## Results

In this section, we will first report the result of our hypothesis test. Subsequently, we will report the results of analyses that explore five further possible explanations for the difference in behavior between the independent and cohesion condition.

### Confirmatory analysis: Group members find the best leader

**Fig 2:**
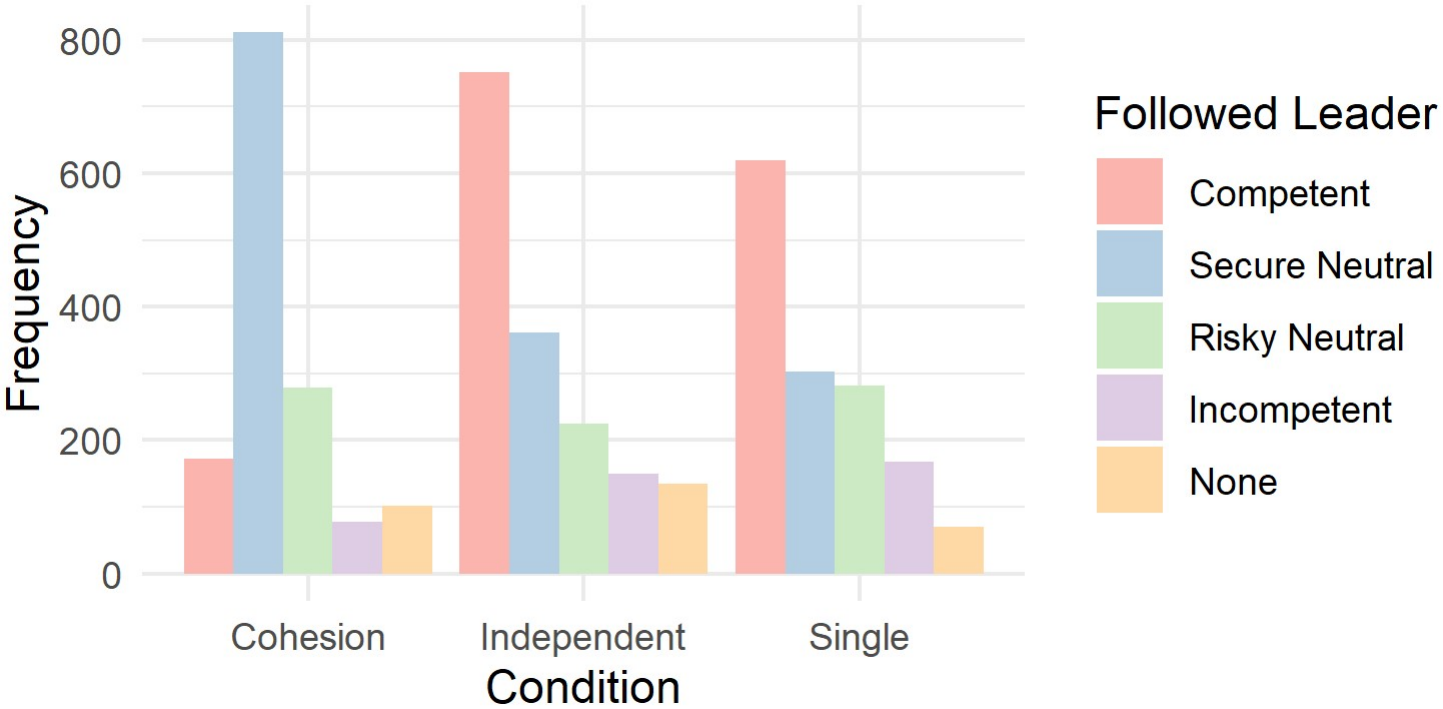
Overview over following behavior according to condition and leader.

As was shown by a Chi-square test, there were significant differences between the frequency with which leaders were followed; *χ*^2^(4) = 1518.3, *p* < .001. Across rounds and conditions, participants followed both the competent and the secure neutral leader more frequently compared to the risky neutral leader (784 times, *p* < .001; p-values corrected for multiple comparisons with Bonferroni method) and the incompetent leader (394 times, *p* < .001), while participants generally followed the competent leader approximately as often (1543 times) as they followed the secure neutral leader (1474 times, *p* = 1). However, Fig 2 shows differences between conditions: Participants in the single and independent conditions followed the competent leader with the highest frequency, while participants in the cohesion condition mostly followed the secure neutral leader.

**Fig 3:**
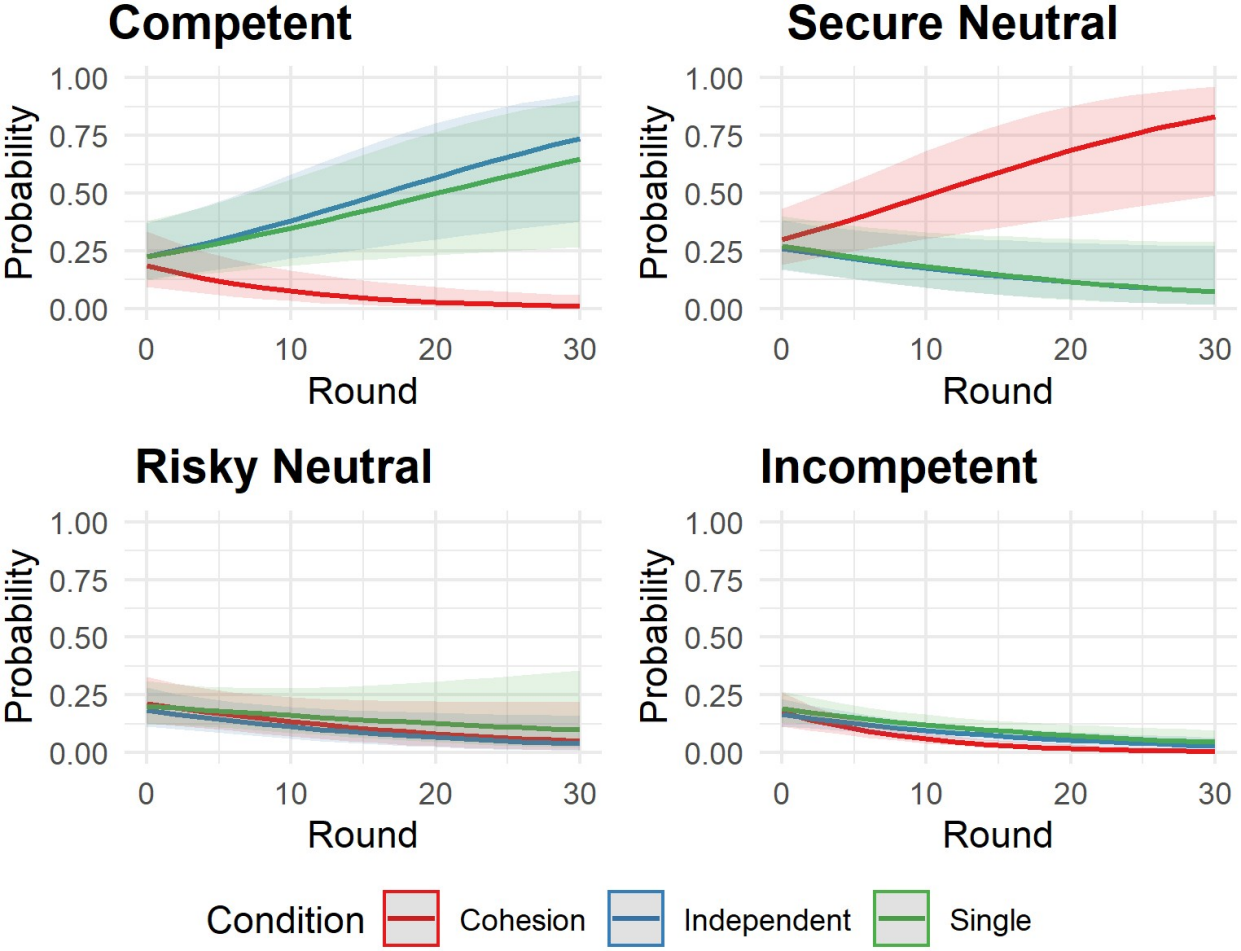
Predicted probability to follow leaders. Plot shows the predicted values for the probability to follow the individual leaders depending on condition and round. Shaded areas represent the 95% C.I.

We fitted a logistic mixed model (estimated using ML and BOBYQA optimizer) to predict the following of the competent leader with condition and round, excluding the intercept (formula: Following of Competent Leader (0 or 1) ~ -1 + condition * round). The model included round, participant id and group as random effects. Participants in the single condition were randomly grouped in pseudo-groups for this analysis. The model’s total explanatory power is substantial (conditional R^2^ = 0.70) and the part related to the fixed effects alone (marginal R^2^) is of 0.21. We fitted the same model for the other three leaders (secure: conditional R^2^ = 0.64, marginal R^2^ = 0.15; risky: conditional R^2^ = 0.53, marginal R^2^ = 0.03; incompetent: conditional R^2^ = 0.36, marginal R^2^ = 0.15). Detailed results on the effects within these models are shown in the S1 Table and Fig 3. From these, it can be observed that the cohesion condition differed in remarkable ways from both the single and independent condition. While in both, the single and independent condition participants showed a significant increase in advantageous decisions over the 30 rounds (following the competent leader) and a decrease in disadvantageous decisions (following the risky or incompetent leader), participants in the cohesion condition showed an increase in mediocre decisions over the 30 rounds (following the secure neutral leader) and a decrease in advantageous decisions (following the competent leader).

To further investigate the key factors behind these observations, we explored further possible explanations. In the following, we will focus on the comparison of the independent and cohesion condition as some of the following analyses would require to form pseudo-groups of separate participants in the single conditions. We believe that statistically created pseudo-groups of separate players in the single condition are not meaningfully comparable to conditions where participants played in real groups and, therefore, choose to focus on the comparison between the cohesion and the independent conditions.

### Exploratory analysis

#### Control for payout realization

Participants were rewarded according to the payout structure of the pre-programmed leaders. Because these were defined via random draws from a binomial distribution, it is possible that unlikely realizations may have caused unexpected rewards for participants. For example, in an unexpected realization the secure neutral leader might have paid out more over 30 rounds than the competent leader. Therefore, we investigated whether games played in the cohesion condition had more unlikely payout realizations compared to those in the independent condition. The leader payout averages over 30 rounds were calculated for all games. On the accumulated level, leader payout realizations were always close to the theoretically expected values as can be seen in the S1 Fig. In no game did we find that the order of leaders in terms of expected payout was changed (sorted from competent to incompetent). Hence, we are able to exclude this as an explanation for our findings.

#### Rewards after leader-change

While we did not find unlikely payout realizations in between games, specific payout realization on a per-round-level might still be present. An especially critical point, where unlikely payout realizations might have a significant effect, is the moment after a participant has changed the leader he/she had followed before. A leader change is identified whenever a participant (a) followed one leader in a given round and this leader was not followed in the subsequent round or (b) followed no leader in a given round and followed any leader in the subsequent round. Random disproportionate punishment or reward of leader changes could then serve as an explanation for the differences observed between the independent and the cohesion condition. Especially, disproportionate punishment of leader changes in the cohesion condition might have discouraged participants in this condition to try out other leaders.

We conducted a Breslow-Day-Test of homogeneity of odds ratios that did not support this explanation: There was no significant difference in the odds ratio between the cohesion (odds ratio = 0.535) and independent condition (odds ratio = 0.594); χ^2^(1) = 0.347, *p* = 0.55. This means that participants in the cohesion and independent condition were equally likely to receive a payout (or not) after a leader change. In fact, it seems as if not receiving a payout after a leader change was less likely than receiving a payout after a leader change as can be seen in Table 1. Hence, we reject this explanation for our data as well.

**Table 1:** Contingency tables for cohesion and independent condition.

		Cohesion		Independent	
Leader Change		Yes	No	Yes	No
Payout	Yes	325	787	508	805
	No	122	158	220	207

Chi-Square Test of Independence showed a significant difference between observed and expected cell values in both cohesion (χ^2^ (1)_Coh_ = 20.46, p < .001) and independent condition (χ^2^(1)_Ind_ = 21.28, p < .001).

#### Exploration and exploitation phases

Participants in the current study might have gone through an initial trial-and-error-phase (exploration) before sticking with the option they believed to have the most advantageous outcome (exploitation). In the exploration phase, participants would be expected to show more frequent leader changes compared to the exploitation phase [27]. Differences between the independent and cohesion conditions could have resulted from the cohesion reward inadvertently shortening the exploration phase of participants in the cohesion condition, as has been shown in previous research [56]. We explore this hypothesis in the following.

First, we checked whether we could identify exploration and exploitation phases in the behavior of participants. To this end, the metric "half-change-round" was defined. The half-change-round is defined to be the round in which at least half of all leader changes of one participant had occurred. For example, within 30 rounds one participant changes the leader 15 times in total. After 10 rounds, this participant has already changed the leader 8 times (15/2 = 7.5, rounded 8). Hence, by round 10, the participant has already performed at least half of all leader changes. Consequently, round 10 would be defined as that player’s half-change-round. It should be noted that the half-change-round does not correspond to the length of the exploration phase as still half of leader changes happen after this point of time.

We found that on average, participants made half the leader-changes after 10 or 11 rounds (half-change-round = 10.82) which, according to a one sample t-test testing (μ = 15), lies significantly below the midpoint of the game at 15 rounds (difference = -4.18, 95% CI [9.70, 11.95], t(107) = -7.33, p < .001; Cohen’s d = -0.71, 95% CI [-0.92, -0.50]), confirming that participants are more likely to change their leader in the early phases of the game. However, when conducting a Welch two-sample t-test, we did not find a significant difference in half-change-rounds between the independent (11.1 rounds) and cohesion condition (10.48 rounds); difference in means = -0.62, 95% CI [-2.94, 1.69], t(94.47) = -0.53, p = 0.596; Cohen’s d = -0.10, 95% CI [-0.49, 0.28]. This means that on average participants in the cohesion and independent condition stayed in the exploration phase equally long, so that this could not explain the difference in following behavior between the conditions.

#### Clustering

We explored whether clustering of the participants might be the cause of the observed effects. Due to the collective reward structure, groups in the cohesion condition might have stayed together as a group throughout the game, thereby inhibiting individual exploration. If this were true, we would expect that clustering on the playfield would at least partially mediate the relationship between condition and following behavior. It should be noted that we understand clustering (“staying in close spatial distance”) as a procedural pattern of group decision making in HoneyComb, comparable to concepts of interdependence in group processes [57] or local information exchange through direct interaction [44]. Based on this, clustering might influence participants behavior on the playfield additional to goal-directed behavior (“finding the best leader”; [58]), for example, as collective feedback [44]. Clustering, therefore, is a variable describing the spatial clustering of participants on the playing field, similar to “flocking” [59], and has no connection to statistical cluster analysis of certain variables of those participants. Clustering is understood as a variable on the group level (global clustering), compared to clustering around a certain participant (local clustering). Hence, clustering does not only take the movement of one individual participant into account, but the movement of the whole group.

We operationalized clustering of the participants as transitivity in a network based on the final movement coordinates of participants and leaders. More information on how the networks were constructed is given in the S5 Text. In this network, the nodes are agents (participants and leaders), while the edges indicate the distance between two participants or a participant and a leader. The strength of the edge between two nodes representing a participant or leader was set to be the inverse of the shortest path between these two (closeness = 1/(shortest path + 1); addition of 1 to prevent division by zero). For example, if Participant A finished the round on the same field as Participant B (shortest path = 0 fields) and within three fields of Participant C (shortest path = 3 fields), the connection (the edge) between A and B would be stronger than the connection between A and C. Specifically, the weight of the edge between nodes A and B would be set to 1 (1/(0 + 1) = 1), while the weight of the edge between A and C would be 0.25 (1/(3 + 1) = 0.25). Transitivity is defined as the overall probability in a network (or graph) that adjacent nodes are interconnected. Under perfect transitivity, if a node A is connected (by an edge) to B, and B is connected to C, then A and C are also connected. High transitivity in a group of HoneyComb players would indicate that its members often moved together throughout the game. In order to reflect our understanding of clustering as a procedural pattern, we calculate a moving average of transitivity over five rounds (transitivity in round 5 (6, 7, …) = mean of transitivity in round 1–5 (2–6, 3–7, …). In doing so, we incorporate the fact that participants’ behavior in a given round is also informed by experience about other participants’ behavior in previous rounds. Further information on how transitivity was calculated can be found in the S5 Text. We also note that this operationalization of clustering is close to the concept of crowd density that is often used in research of moving human groups [60–62].

We did not find a significant difference in transitivity between the independent and cohesion conditions when conducting a Welch two-sample t-test between the mean transitivity in the cohesion and independent condition; t(100.77) = 0.00, p >.999. This means that even though cohesion was not incentivized in the independent condition, participants often moved closely together as had already been observed in previous HoneyComb experiments [59]. We further explored the effects of condition, transitivity, and round on following behavior (i.e., which leader a participant followed) by fitting a logistic mixed model (estimated using ML and BOBYQA optimizer) to predict following the competent leader with condition, round and transitivity (formula: Following the competent leader (0 or 1) ~ condition * round * transitivity). The model included round, participant id and group as random effects. The model’s total explanatory power is substantial (conditional R^2^ = 0.76) and the part related to the fixed effects alone (marginal R^2^) is of 0.25. In order to identify whether the additional inclusion of transitivity as an explanatory model increases model fit, we compared this model to a model including only condition and round as explanatory variables. Both models were then compared using the likelihood ratio test. We repeated this procedure for the other three leaders (secure: conditional R^2^ = 0.72, marginal R^2^ = 0.17; risky: conditional R^2^ = 0.62, marginal R^2^ = 0.04; incompetent: conditional R^2^ = 0.36, marginal R^2^ = 0.08). The complete results can be seen in Table 2.

**Table 2:** Results of logistic regression model of probability to follow different leaders.

Predictors	Beta (SE)	p	Overall model^a^
Competent			
Condition^b^	7.66 (3.06)	**.012**	χ^2^(4) = 9.91 p = .**042**
Round	0.16 (0.15)	.291	
Transitivity	8.10 (3.40)	**.017**	
Condition x Round	-0.20 (0.18)	.272	
Condition x Transitivity	-9.59 (4.03)	**.017**	
Round x Transitivity	-0.35 (0.20)	.078	
Condition x Round x Transitivity	0.49 (0.23)	**.035**	
Secure Neutral			
Condition^b^	-4.49 (2.70)	.096	χ^2^(4) = 14.22 p = .**006**
Round	0.019 (0.11)	.865	
Transitivity	2.85 (2.44)	.243	
Condition x Round	0.21 (0.15)	.179	
Condition x Transitivity	3.76 (3.51)	.283	
Round x Transitivity	-0.00 (0.14)	.991	
Condition x Round x Transitivity	-0.36 (0.20)	.077	
Risky Neutral			
Condition^b^	-5.83 (2.70)	**.031**	χ^2^(4) = 19.66 p < .**001**
Round	-0.28 (0.13)	**.032**	
Transitivity	-11.90 (3.00)	**< .001**	
Condition x Round	0.17 (0.17)	.325	
Condition x Transitivity	10.41 (3.76)	**.006**	
Round x Transitivity	0.43 (0.18)	**.015**	
Condition x Round x Transitivity	-0.38 (0.22)	.092	
Incompetent			
Condition^b^	0.42 (3.58)	.906	χ^2^(4) = 2.45 p = .653
Round	0.14 (0.21)	.498	
Transitivity	2.27 (3.99)	.569	
Condition x Round	-0.10 (0.24)	.677	
Condition x Transitivity	0.50 (4.84)	.918	
Round x Transitivity	-0.26 (0.28)	.352	
Condition x Round x Transitivity	0.14 (0.32)	.656	

For all models, the outcome variable was set to 1 when a participant had arrived at a specific leader and set to 0 when he/she had arrived at a different or no leader. The explanatory variables were condition (independent vs. cohesion), round, and transitivity, as well as their interactions. Playgroup, participant id and intercept were included as fixed effects but are not reported here. Round was included as a random effect.

^a^Model selection for inclusion of transitivity in the model based on likelihood ratio test.

^b^Cohesion condition was dummy coded 0, independent condition as 1.

**Fig 4:**
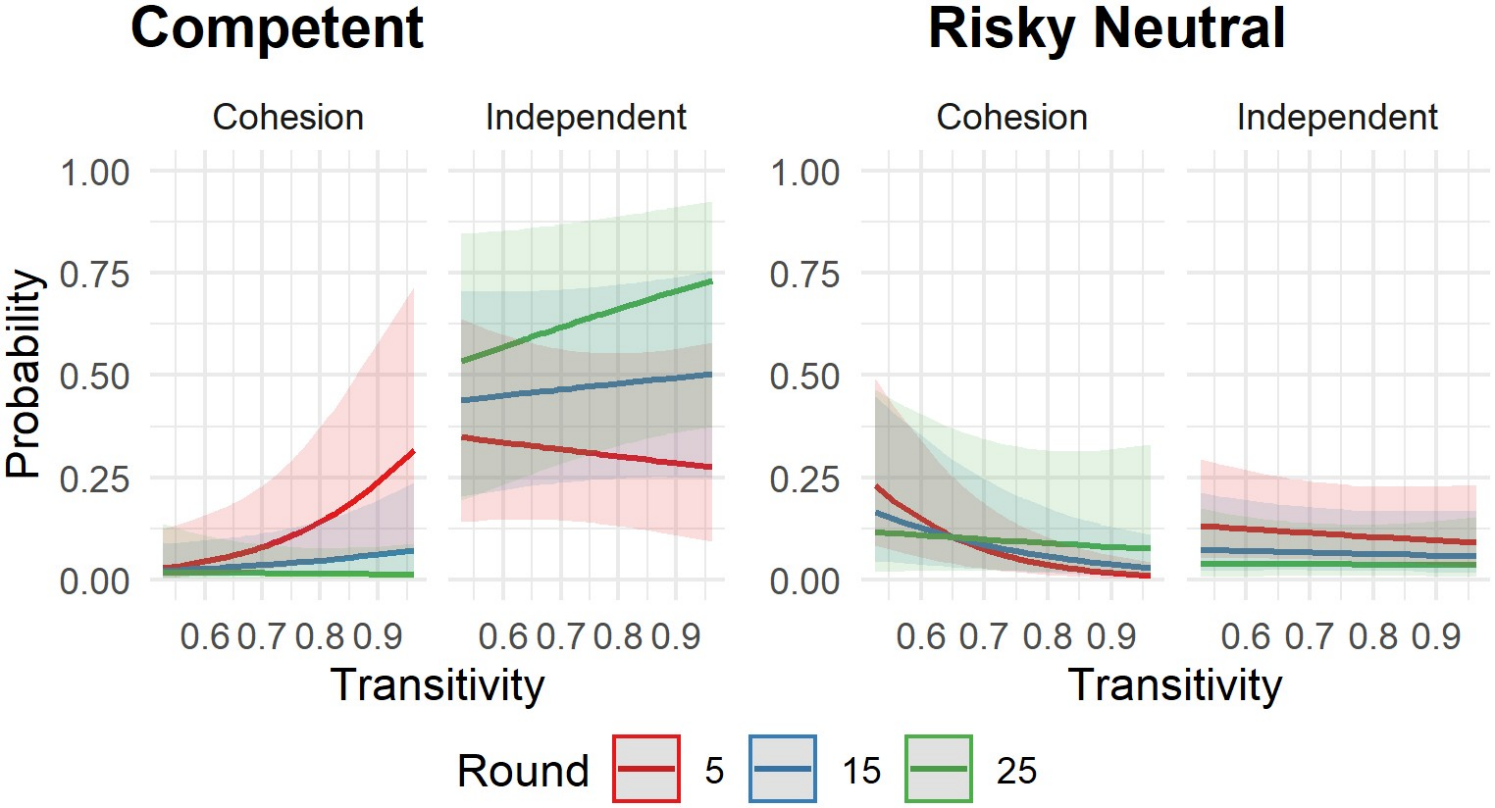
Predicted probability to follow competent or risky leader. Plot shows the predicted values of the probability to follow either the competent or risky neutral leader in relation to condition, round, and transitivity. Shaded areas represent the 95% C.I. Round 5, 15 and 25 represent an early, middle, and late round in the game, respectively.

On the one hand, neither condition, round, nor transitivity had any effect on how likely participants were to follow the secure neutral or the incompetent agent. On the other hand, we found that participants were more likely to follow the competent leader in the cohesion condition when transitivity was high during early rounds (*b* = 0.49, *p* = .035), as can be seen in Fig 4. During later rounds, the effect of transitivity ceased. The opposite was true for participants in the independent condition: During the early rounds, they were less likely to follow the competent leader if transitivity was high. During later rounds, however, participants in the independent condition were more likely to follow the competent leader if transitivity was high. For the risky leader, we found that participants in both conditions were more likely to follow during early rounds and when transitivity was low (*b* = 0.43, *p* = .015).

The explanatory value of transitivity is further corroborated by the fact that in three out of four models (competent, secure neutral, and risky neutral leader), the inclusion of transitivity improved the model fit significantly (see Table 2). In order to check whether this additional explanatory variable was due to a mediation effect, we conducted a mediation analysis, including the explanatory variables condition and round, the mediator transitivity, and the outcome variable of following a leader. We did not find any significant mediation effects for any of the leaders. For the purpose of readability of this manuscript, we report the detailed information of the fitted models as well as results of these analyses in the S6 Text.

In sum, transitivity seems to play a role in group decision making as it adds explanatory value to our analyses. However, the results on transitivity should be interpreted with caution as they remain correlational and the direction of influence between the outcome variable, the decisions to follow a leader, and transitivity remain unclear. This will be discussed in further detail in the Discussion section. It seems clear, however, that transitivity does not fully account for the differences in following behavior between the independent and cohesion condition.

#### Reward structure

Finally, we explored whether the reward structure itself might have driven the difference between the independent and the cohesion conditions. Since the payout participants received was their main source of information about the leaders, there should be a direct impact on the participants’ behavior. To accurately identify the best leader, participants would have needed precise information on the leader’s payout properties. However, in the cohesion condition, rewards were multiplied by the number of participants who received it. Thus, participants in the cohesion condition might not have received adequate feedback as the number of participants following one leader and, therefore, the multiplication factor of the rewards could have varied strongly–especially during the exploration phase.

To operationalize the decision behavior of a participant throughout the game, we calculated participants’ choice score using a point system: For each round in which a participant followed the competent leader, two points were awarded. For rounds in which participants followed the neutral leaders (secure or risky), the participants were awarded one point, while receiving zero points for following the incompetent or no leader. The sum of all points over 30 rounds was then set to be the participant’s choice score. We argue that if participants did receive accurate feedback through rewards, payout should only depend on the participants’ behavior (measured by the choice score) and not the condition. However, if the reward structure occluded crucial information from participants in the cohesion condition, we should see a moderation effect of condition on the effect of choice score.

To test this explanation, we fitted a linear mixed model (estimated using REML and nloptwrap optimizer) to predict earnings (corrected for initial endowment) with condition and choice score (formula: earnings ~ condition * choice score). The model included group as random effect. The model’s total explanatory power is substantial (conditional R^2^ = 0.88) and the part related to the fixed effects alone (marginal R^2^) is of 0.80. The model’s intercept, corresponding to condition = Cohesion and choice score = 0, is at 809.10 (95% CI [579.01, 1039.19], t(102) = 6.89, p < .001). Within this model, the effect of condition [Independent] is statistically significant and negative (beta = -958.30, 95% CI [-1240.75, -675.84], p < .001; Std. beta = -1.85, 95% CI [-2.16, -1.53]), showing that condition did have an unintended effect on payout and, therefore, on the feedback participants received from the leaders they followed. The effect of choice score is statistically non-significant (beta = -0.04, 95% CI [-8.22, 8.13], p = 0.992). The interaction effect of choice score on condition [Independent] is statistically non-significant (beta = 7.36, 95% CI [-1.82, 16.53], p = 0.116).

Additionally, we calculated two Pearson’s product-moment correlation tests between earnings (corrected for initial endowment) and choice score separately for the independent and the cohesion condition. For the independent condition, the correlation is positive, statistically significant, and very large (r = 0.87, 95% CI [0.79, 0.92], t(58) = 13.37, p < .001), while statistically not significant and tiny for the cohesion condition (r = -0.03, 95% CI [-0.31, 0.26], t(46) = -0.20, p = 0.840). We note that these correlations should be interpreted with care as they do not include the nesting of participants within their groups.

**Fig 5:**
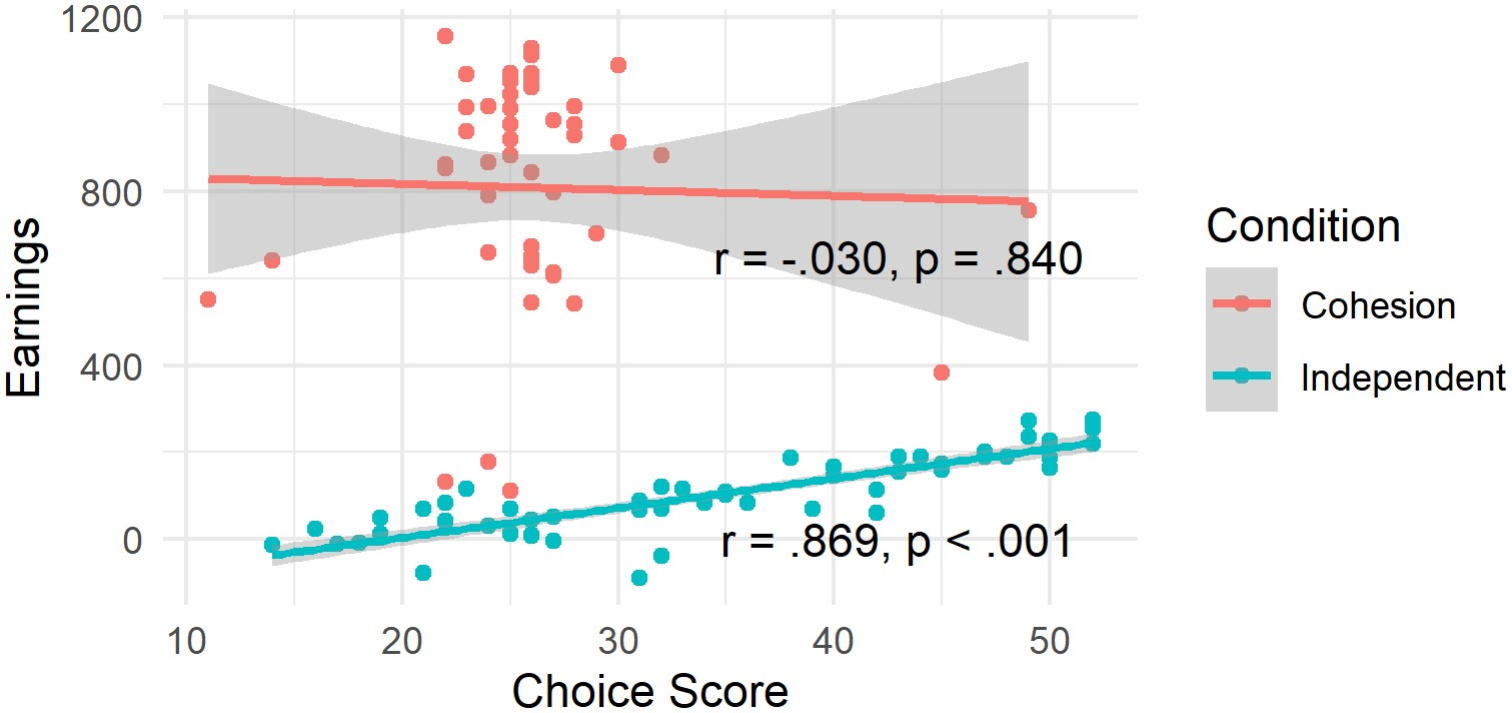
Earnings of participants according to condition and choice score. In the cohesion condition, choice score and earnings (corrected for initial endowment) do not correlate, while in the independent condition, there exists a strong relationship between earnings and choice score. The plot shows the choice score and earnings after 30 rounds, corrected for initial endowment.

These findings fit well to the relationship between choice score and earnings as shown in Fig 5. While we see a very close relationship in the independent condition, the data in the cohesion condition are much more scattered showing a high range in earnings but a comparatively low range in choice score. While it is surprising that this was not represented in a significant effect in the linear model, we can conclude that the reward structure might have interfered with the participants’ estimation of who the best leader could be.

## Discussion

In this study, we used an adapted version of the HoneyComb paradigm [45,50] to investigate whether participants are able to identify the most advantageous of four leaders by gathering information through following and observing others follow these leaders. Additionally, we investigated under which circumstances participants are able to find the best leader: alone or in a group, as independent group members or in a group that rewarded cohesion.

Our results show that group members who repeatedly interact with different leaders are indeed able to identify the leader that is most beneficial to themselves and the group. This holds true, even if they do not have any prior knowledge about the leaders’ abilities but need to infer them from their experience. This is consistent with studies showing that the ability to find the best possible leader in a group might have been a crucial evolutionary advantage [12,14,16].

Apart from this general result, this study shows the impeding effects different circumstances might have on group members. While participants who played alone and participants who played as independent group members followed the most advantageous leader, participants who played in a group that was rewarded for cohesive behavior frequently followed a less advantageous leader. To further explore the reasons for this difference, we conducted a series of exploratory analyses.

The aim of our initial analysis was to rule out confounding factors as explanations for the data observed in the experiments. Our results show that there was no systematic difference between the cohesion and independent condition in the realization of either leader payouts or random punishments of leader changes through non-payouts in the subsequent round. Therefore, we conclude that we can exclude these confounding factors as suitable explanations for the differences between the independent and cohesion condition.

Next, we investigated the possibility that participants in the cohesion condition might have exhibited a shorter exploration phase, compared to participants in the independent condition, as was suggested by previous research [56]. To this end, we developed the metric of the half-change-round to operationalize the length of both exploration and exploitation phases in the HoneyComb paradigm. Our results show that in both the independent and cohesion condition participants first explore the behavior of different leaders and, afterwards, exploit the leader they believe to be best. However, our results do not show shorter exploration phases for the cohesion condition. This is surprising as we expected that a more cohesive group might suppress the gathering of private information for the benefit of cohesive behavior as suggested by previous studies on group decision making [56], hidden profiles [63], or groupthink [64]. We believe that this area is an interesting subject for further investigation, aided by the newly developed half-change-round metric.

In an attempt to explain the surprising effects, we then investigated the influence of clustering, a measure of how closely group members stick together, on a group’s following behavior. Our results show that clustering does play a role for an individual’s ability to identify the most advantageous leader. For independent group members, a high clustering seems to be disadvantageous during early phases, but advantageous during later phases. The opposite seems to be true for groups with a high incentive to act cohesively: High clustering is advantageous during early phases but has no effect later on. This might be explained in the following way: When no pressure to act cohesively exists, participants are free to try out different and also less advantageous leaders in the beginning, gathering private information. When group members learn who the most advantageous leader is, others can learn from them, thereby integrating private information on the collective level [44]. When more individuals follow one leader, others are reinforced to do the same, creating a positive reinforcement loop that has been observed in collective animal movement and crowd behavior [44]. In this manner, more and more group members join into the cluster, following the best leader. However, when acting cohesively is incentivized, as in the cohesion condition, clustering might not be a result of individual decisions but rather the basis on which group decisions are made [56]. As a result, a group might cohesively explore some leaders during early phases but the one they settle on in the exploitation phase might not be the most advantageous one. This explanation is in line with studies which show that a high emphasis on group cohesion can be detrimental to group decision making [56], a prominent example being groupthink [65].

Contrary to this explanation, the effect of condition on the ability to find the best leader was not significantly mediated by clustering. This might be due to the effect that no meaningful difference in clustering was found between conditions. We could explain this in two ways: First, other HoneyComb studies have found that participants generally exhibit high levels of clustering on the playfield, even if they are not instructed or rewarded to do so [59]. Second, we cannot draw definite conclusions about the causal direction of the relationship between clustering and following behavior. We assume that participants in the cohesion condition moved as a group because they were incentivized for this behavior and that clustering had an effect on the participants’ ability to find the best leader. Alternatively, it could be argued that the clustering was a side-effect of multiple participants following the same leader. This could explain why groups in the independent condition also exhibited high levels of clustering. Multiple participants independently identified the competent leader as the most advantageous option and then continuously followed that leader. Through repeatedly following the same leader, participants inadvertently also moved as a group. Because of the correlational nature of our data on this relationship, we cannot rule out either explanation.

However, as we did not find consistent effects of transitivity on following behavior towards the different leaders, we do not believe that this can explain the remarkable difference between the cohesion and independent groups. Therefore, we investigated whether the reward structure might be the driving force behind these results. The results seem to substantiate this explanation: The financial outcome is not influenced by the participants’ following behavior, but rather by the membership in either the independent or cohesion condition. Additionally, we might have expected a moderator effect of condition because participants in the independent condition received unadulterated monetary feedback from the leaders, while the monetary feedback given to participants in the cohesion condition was occluded by the multiplication of rewards. We did not find support for this in the linear model. However, we could show in a further exploratory analysis that choice score and earnings did highly correlate in the independent condition, while they did not correlate in the cohesion condition. We note that these correlations do not take nesting of data into account so that they should serve solely as an illustration while we rest the following conclusions on the results of the linear model. From it, we assume that in the current experiment participants’ earnings were mainly determined by experimental condition and not participants’ decision behavior. This is problematic as the financial outcome is the central feedback that participants can receive from the leaders they followed and, therefore, the basis of their estimation of which leader is most advantageous. If this source of information is faulty (i.e., not reflective of the individuals’ behavior) then participants are deprived of the only way to make an informed decision about the leaders. Our results show that the reward structure we implemented–multiplying a reward by the number of group members on the same reward field to encourage cohesive action–has likely occluded the information participants need to make an informed decision. We believe that this information was occluded, but not inaccessible altogether as, theoretically, participants in the cohesion condition could have been able to infer the differences between the leaders. As can be seen in the S2 Table, participants in the cohesion condition might have earned less when they followed the competent leader alone, compared to when following the secure leader with other participants. However, they would have earned even higher payouts when following the competent leader. Thus, participants should have been able to infer the best leader, albeit it might have been more difficult (participants would have to divide the reward by the number of co-players on the field to get a better estimate). Moreover, participants in the cohesion condition might have felt satisfied with the high rewards they earned by following less advantageous leaders, thereby reducing the need to gather more information about the other leaders, which in the end cost them the knowledge of the most advantageous outcome.

Lastly, we want to draw attention to the fact that humans are a species, naturally living in groups [12]. As such staying together as a group throughout the game might be rewarding in and of itself. We find support for this argument in the fact that participants in the independent condition clustered about as much as participants in the cohesion condition. This conforms with findings from previous experiments in the HoneyComb paradigm in which participants were found to move relatively cohesively (“flock”) even though they did not receive any special instruction to do so [59].

### Implications and conclusion

Our findings show that group members are able to find the most advantageous leader by exploring different possible leaders and later exploiting the one they believe to be best. We show that these results are observable in a reductionist paradigm like HoneyComb [45,50] that allows researchers to control the experimental environment closely and even remove most of the communication channels that are typically used in group interaction (visual and auditory verbal and nonverbal communication), so that participants can only communicate through movement.

Furthermore, we developed a new metric to quantify and compare the length of exploration and exploitation phases in group decision making: the half-change-round. We note that while other scientific fields may have used similar constructs to quantify phases (e.g., transition phases in physical systems), the half-change round constitutes, to the authors’ knowledge, the first application of such an approach to quantify the phases of a group decision making process. Using this tool, we could show that group decision settings can be divided into an exploration and exploitation phase.

Lastly, our results shine a spotlight on a number of confounding factors in group experiments, including reward structures that are used in order to create certain group behaviors. In this paper, we present ways to check for and either identify or exclude them as alternative explanations for results of group experiments. We specifically want to draw attention to the setup of reward structures in group experiments. We argue that researchers need to take a close look into how reward structures might inadvertently affect the phenomena that researchers wish to investigate. In our case, creating cohesion through multiplication of rewards impeded participants in the cohesion condition in their gathering of essential information, thereby influencing behavior directly rather than through the creation of a cohesive group behavior. Retrospectively, a more suitable option than multiplication of rewards might have been to create cohesion through a shared group account or fixed group bonus.

As a result of this, we believe that while some of the mentioned results were driven by the chosen reward structure, other results (e.g., length of exploration phases or effect of transitivity) might be partially occluded by the strong influence of reward structure on behavior. Hence, we suggest that the concept of differing exploration and exploitation phases, as well as the influence of clustering warrant further investigation in future research. In doing so, researchers should be especially careful when choosing a reward structure to not cost their participants the best behavior, by giving them rewards “for free”.

## Supporting information

S1 Fig Overview of payout realization for all games played. This overview includes the averages of all payouts leaders made during a game, regardless of condition and whether the leaders were followed.
(PNG)

S1 Table Results of logistic regression model of probability to follow different leaders. For each leader type, a separate generalized logistic mixed-effect regression was estimated. The probability to arrive at that leader were set to be the outcome variable; condition and round were included as explanatory variables. Participants in the single condition were put into pseudo-groups for comparison. The intercept was excluded in this model. Round was included as a random effect and group and participant ID were included as grouping variables. Numbers in the table are parameter estimates (standard errors in parentheses). *p < .05; **p < .01; ***p < .001.
(DOCX)

S2 Table Interactive table of payout matrix. (XLSX)

S1 Text Detailed description of technical setup and game software. (PDF)

S2 Text Additional information on experimental procedure. (PDF)

S3 Text Summary of questionnaires. (PDF)

S4 Text List of used R-packages. (PDF)

S5 Text Description of how networks and transitivity were computed. (PDF)

S6 Text Detailed information on mediation analysis. (PDF)
