# Supplementary figures and images for: How collective reward structure impedes group decision making: An experimental study using the HoneyComb paradigm
Source: PLoS One. 2021 Nov 16;16(11):e0259963. doi: 10.1371/journal.pone.0259963 (PMC8594797; doi:10.1371/journal.pone.0259963)

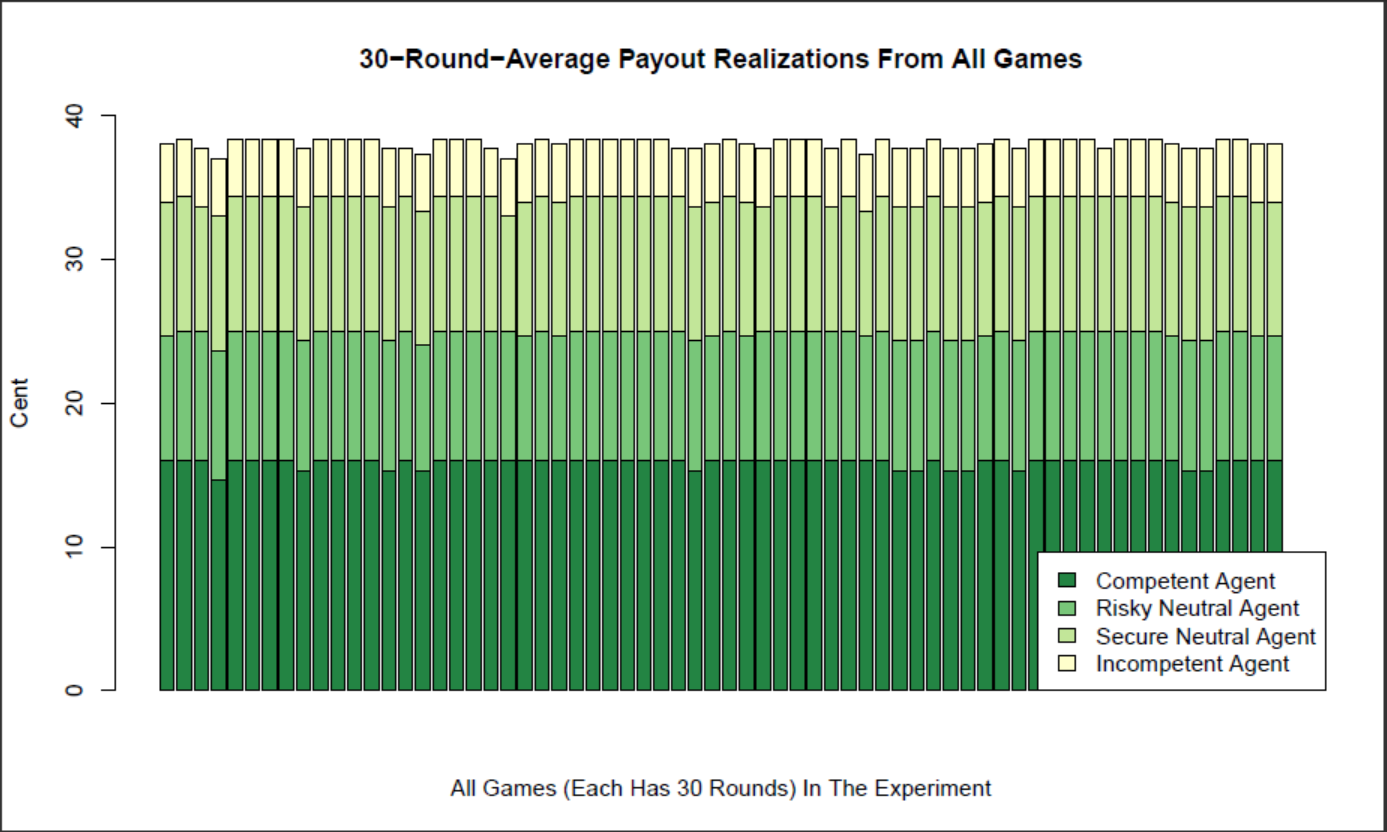

Supplement: S1 Fig — This overview includes the averages of all payouts leaders made during a game, regardless of condition and whether the leaders were followed. (PNG) [file pone.0259963.s001.png]
