# Supplementary material for: How collective reward structure impedes group decision making: An experimental study using the HoneyComb paradigm
Source: PLoS One. 2021 Nov 16;16(11):e0259963. doi: 10.1371/journal.pone.0259963 (PMC8594797; doi:10.1371/journal.pone.0259963)
Supplement: S1 Table — For each leader type, a separate generalized logistic mixed-effect regression was estimated. The probability to arrive at that leader were set to be the outcome variable; condition and round were included as explanatory variables. Participants in the single condition were put into pseudo-groups for comparison. The intercept was excluded in this model. Round was included as a random effect and group and participant ID were included as grouping variables. Numbers in the table are parameter estimates (standard errors in parentheses). *p < .05; **p < .01; ***p < .001. (DOCX) [file pone.0259963.s002.docx]

**S5 Table. Results of logistic regression model of probability to follow different leaders.**

**Table S5**

Results of Logistic regression model of probability to follow different leaders

| **Predictors** | **Beta (SE)** | **p** |
| --- | --- | --- |
| Competent leader | | |
| Single condition | -1.26 (0.39) | **.001** |
| Independent condition | -1.50 (0.42) | **< .001** |
| Cohesion condition | -1.15 (0.39) | **.003** |
| Round | -0.10 (0.03) | **< .001** |
| Round * Cohesion | 0.18 (0.04) | **< .001** |
| Round * Single | 0.16 (0.04) | **< .001** |
| Secure neutral leader | | |
| Single condition | -1.01 (0.32) | **.001** |
| Independent condition | -1.08 (0.32) | **< .001** |
| Cohesion condition | -0.85 (0.32) | **.008** |
| Round | 0.08 (0.02) | **< .001** |
| Round * Cohesion | -0.13 (0.03) | **< .001** |
| Round * Single | -0.12 (0.03) | **< .001** |
| Risky neutral leader | | |
| Single condition | -1.35 (0.28) | **< .001** |
| Independent condition | -1.57 (0.29) | **< .001** |
| Cohesion condition | -1.31 (0.29) | **< .001** |
| Round | -0.05 (0.03) | .051 |
| Round * Cohesion | 0.00 (0.03) | .951 |
| Round * Single | 0.03 (0.03) | .483 |
| Incompetent leader | | |
| Single condition | -1.42 (0.23) | **< .001** |
| Independent condition | -1.66 (0.24) | **< .001** |
| Cohesion condition | -1.56 (0.26) | **< .001** |
| Round | -0.12 (0.02) | **< .001** |
| Round * Cohesion | 0.06 (0.02) | **.011** |
| Round * Single | 0.06 (0.02) | **.008** |

*Note.* For each leader type, a separate generalized logistic mixed-effect regression was estimated. The probability to arrive at that leader was set to be the outcome variable; condition and round were included as explanatory variables. Participants in the single condition were put into pseudo-groups for comparison. The intercept was excluded in this model. Round was included as a random effect and group and participant ID were included as a grouping variables. Numbers in the table are parameter estimates (standard errors in parentheses). Standardized parameters were obtained by fitting the model on a standardized version of the dataset. 95% Confidence Intervals (CIs) and p-values were computed using the Wald approximation.

*p<.05; **p<.01; ***p<.001
