# Supplementary material for: How collective reward structure impedes group decision making: An experimental study using the HoneyComb paradigm
Source: PLoS One. 2021 Nov 16;16(11):e0259963. doi: 10.1371/journal.pone.0259963 (PMC8594797; doi:10.1371/journal.pone.0259963)
Supplement: S1 Text — (PDF) [file pone.0259963.s004.pdf]

S1. Detailed description of technical setup and game software

12 laptops of similar size (screen size 13.3 in. with one screen of 12.8 in.) and identical resolution quality (1366 × 768 pixels) were used. These laptops were all connected via a common server and placed at individual workstations separated by partition walls (Figure S1). At the workstations, participants were provided with a mouse to navigate the game and earplugs to restrict communication throughout the experiment. Furthermore, at their workstations, participants were provided with a pen, a form of consent and abbreviated versions of the Iterated HoneyComb Game rules for both roles of the game (informed vs. non-informed) in addition to the computerized instructions displayed on the laptop screens.

Figure S1

Experimental Setup

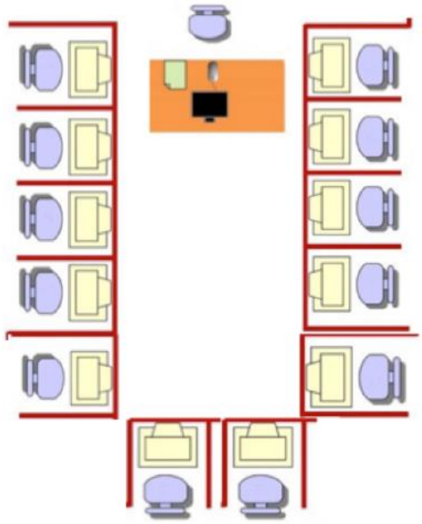

Developed and maintained by Johannes Pritz, the application software of the HoneyComb© games are Java-based and often custom-made (i.e., adapted to different experimental setups). Our pilot study made use of two versions of the game that were programmed in 2010 and 2016, respectively. Both versions of the software displayed their computerized instructions prior to the game. The first version of the game served as a practice round and helped the participants to familiarize themselves with the gaming environment. The second version of the game was the Iterated HoneyComb Game. This game included additional features, such as the movement behavior of the four different computed leaders,

the placement of each leader's target fields that either yielded a reward or not and algorithms for computing earnings and payments. As a general rule, computed leaders moved their cursor 3000 ms before movement was enabled for human players. They moved with a randomly chosen velocity (i.e., frequency at which leaders moved) ranging from 1200 ms and 3000 ms. Computed leaders always took the shortest possible path toward their target field by following an algorithm for distance reduction. The computed leaders' target fields were always at the edge of the virtual playing field except for those hexagon fields that formed a corner of the honeycomb. Consequently, computed leaders' paths toward their target field were never purely linear and comprised of exactly seven steps. Target fields were programmed to lie at a minimum angle of 60° apart from each other while

occupying the same position alongside different bordering lines of the hexagon. These bordering lines were randomly chosen for each computed leader at the start of every round of the game. Similarly, a computer algorithm randomly chose if the target field was rewarding for a particular round of the game. Throughout the 30 rounds of the game, rewarding properties of target fields accumulated to the expected reward values of the computed leader. When the target field was non-rewarding, another algorithm randomly led computed leaders either to arrive at the target field or to stop one or two steps in front of it. Real players' reception of reward occurred when they followed computed leaders to their target field during a round of the game when the target field was rewarding. The reward magnitude was set to remain constant for each computed leader (20 cents vs. 10 cents). The same goes for the payoff probability throughout the 30 trials (80% vs. 90% vs. 45% vs. 20%). Human players incurred costs of 1 cent for every step they took following one leader (i.e., directly moving behind or parallel with a leader. Total costs can amount to a maximum of 8 cents since the human players were provided with a budget of eight steps in each round of the game. Gains, losses, and the total amount of money on the account were computed for each player during each round of the game and displayed at the upper left corner of the playing field. Additionally, this information was displayed as a feedback at the end of the game. Furthermore, the top left corner of the playing field displayed a timer set to 30 seconds, which was the time players had to complete one round of the game.

Game Graphics. To ensure that the differently colored avatar dots were distinguishable for all players, we used a set of colors that is unambiguous for both color blind and non-color blind people (Okabe & Ito, 2008) and ensured a minimum contrast ratio of 3:1. For the computed leaders, in particular, we applied four colors that are found to be most easily identifiable regardless of color perception ability (Ichihara et al., 2001) and counterbalanced these four colors for the computed leaders across players and groups.

References for S1

Ichihara, Y. G., Okabe, M., Iga, K., Tanaka, Y., Musha, K., & Ito, K. (2008). Color universal design: The selection of four easily distinguishable colors for all color vision types. *Color Imaging XIII: Processing, Hardcopy, and Applications*, *6807*, 68070O.

Okabe, M., & Ito, K. (2008). Color universal design (cud)-how to make figures and presentations that are friendly to colorblind people. *Retrieved April*, *21*, 2017.
