# Supplementary material for: How collective reward structure impedes group decision making: An experimental study using the HoneyComb paradigm
Source: PLoS One. 2021 Nov 16;16(11):e0259963. doi: 10.1371/journal.pone.0259963 (PMC8594797; doi:10.1371/journal.pone.0259963)
Supplement: S2 Text — (PDF) [file pone.0259963.s005.pdf]

S2. Additional Information on Experimental Procedure

Oral instructions. After Participants gave their informed consent, they were jointly told that they played a multi-client game as a group and therefore had to expect waiting times while playing the game.

Practice Round. Before the begin of the Iterated HoneyComb Game players were instructed to familiarize themselves with the visual environment in a practice round. Thus, all players were introduced to and required to move their avatar in the presence of other players across the virtual playing field. By doing so, they experienced that each of their movements was delayed by a short duration (500 ms) and that they could only move to an adjoining field. Players also learned that their perception of the virtual field was restricted to the fields within a radius of two hexagonal fields surrounding their avatar. Furthermore, they got to know that the small tail appearing next to the avatar for a small duration after the execution of a move (4000 ms) indicated the direction of movement of that avatar. During this practice round, the number of the players on the field was equal to the number of human players participating in two parallel games at the same time. For the independent and cohesion condition condition, a total of 12 players participated in the practice round. In the single condition, only two players were present in the practice round. The practice round required players to execute a total of eight steps and ended after every player on the playing field had completed their eight steps.

Iterated Honeycomb Game. After detailed instructions that explain the rules of the Iterated Honeycomb Game and the roles therein, a number of rounds were played by the participants. Each round of the game began with the computed leaders moving first and ended after 30 seconds had elapsed. Similar to the practice round, players total number of steps for each round of the game was restricted to eight. Players had to pay 1 cent whenever they exhibited one of the following behaviors: directly following a leader, following the leader on a parallel course, surpassing the leader and heading towards the same direction.
