# Supplementary material for: How collective reward structure impedes group decision making: An experimental study using the HoneyComb paradigm
Source: PLoS One. 2021 Nov 16;16(11):e0259963. doi: 10.1371/journal.pone.0259963 (PMC8594797; doi:10.1371/journal.pone.0259963)
Supplement: S3 Text — (PDF) [file pone.0259963.s006.pdf]

S3. Summary of Questionnaires

Risk propensity. R1. R1 represents a single self-report item. Players are asked to indicate their general willingness to take risks on a seven-point scale from “not at all willing to take risks” to “very willing to take risks". Over an average of a six-week period, a test-retest reliability of .74 was found for this item (Beierlein, Kovaleva, Kemper, & Rammstedt, 2014). R1 exhibits a robust convergent validity with the sensation-seeking subscale ($r = .69 - .77$, $p < .001$) of the Urgency Premeditation Perseverance and Sensation Seeking Impulsive Behavior Scale (Whiteside & Lynam, 2001).

HPI-R. The HPI-R is a subscale of the Hamburg personality inventory (HPI) developed by Andresen (1995) and measures self-assessed risk aptitude (R) concerning a relatively stable personality factor in additions to the Big Five. It has been reported to correlate with several external criteria (Berth & Brähler, 2002). The HPI-R subscale consists of 14 positively phrased items that are answered on a 4-point scale from “completely true” to “completely false”. Test-retest reliability within a 1.5-year interval and internal consistency (Cronbach's α) were reported to be .86 and .85, respectively (Berth & Brähler, 2002). For the current sample, Cronbach's α yields a robust value of .80.

Motor impulsivity. Motor impulsivity is a subscale of the short version Barratt Impulsiveness Scale (BIS-15; Spinella, 2007). As such, it measures the tendency to act on the spur of the moment. It consists of five positively phrased items that are scored on a four-point scale ranging from rarely/never” to “almost always/always”. Internal consistency was evaluated as good for the complete German BIS-15 scale (Cronbach's α = .81). For the current sample, Cronbach's α was acceptable for motor impulsivity (Cronbach's α = .77).

Self-esteem. To assess Self-esteem, a subscale (Emotional Self-esteem) of the Multidimensional Self-esteem (Schütz, Rentzsch, & Sellin, 2006) was used. It consists of seven mostly negatively phrased items on a seven-point scale ranging from “absolutely not/never” to very/always”. Three of the seven statements were related to the intensity of attitudes and four addressed the frequencies of thoughts. Internal consistency Cronbach’s α was .88 estimated on data from the current sample. This internal consistency is in alignment with previously reported values. (Cronbach’s α = .85; Schütz, Rentzsch, & Sellin, 2006).

Embodiment. Based on a bachelor thesis of Heemeyer (2006) we included three items that tested for the player’s degree of identification with their avatars, their social perception of others’ avatars and an estimation of others’ social perception of their avatar. To draw meaningful conclusions from our experiment, it was important to estimate the degree to which the Iterated Honeycomb Game was perceived as a social context. The items were rated on a five-point scale and displayed an internal consistency of .41 (Cronbach’s α) for the current sample.

Additional questions. Additional questions consisted of basic demographic questions and tested for the participant’s previous participation in another Honeycomb experiment, their ability to understand the instructions, and their color perception ability. We also included four questions on a seven-point scale that assessed the player’s perceptions and beliefs about learning in a reward-maximizing manner. Furthermore, they had to indicate if they believed that they or their group exhibited a preference to follow one particular informed player.

References for S3

Andresen, B. (1995). Risikobereitschaft (R)-der sechste Basisfaktor der Persönlichkeit: Konvergenz multivariater Studien und Konstruktexplikation. *Zeitschrift Für Differentielle Und Diagnostische Psychologie*, *16*(4), 210–236.

Beierlein, C., Kovaleva, A., Kemper, C. J., & Rammstedt, B. (n.d.). *Eine Single-Item-Skala zur Erfassung von Risikobereitschaft:* 28.

Berth, H., & Brähler, E. (2003). HPI. Hamburger Persönlichkeitsinventar (Testrezension). *Diagnostica*, *49*, 188–191.

Heemeyer, J. (2006). *Die virtuelle Verkörperung der Identität—Eine literarische Analyse zur Avatar-Identifikation unter besonderer Berücksichtigung des Embodiment- und Immersions-Effektes*. Georg-August-Universität Göttingen.

Schütz, A., Rentzsch, K., & Sellin, I. (2006). *Multidimensionale Selbstwertskala: MSWS; Manual*.

Spinella, M. (2007). Normative data and a short form of the Barratt Impulsiveness Scale. *International Journal of Neuroscience*, *117*(3), 359–368.

Whiteside, S. P., & Lynam, D. R. (2001). The five factor model and impulsivity: Using a structural model of personality to understand impulsivity. *Personality and Individual Differences*, *30*(4), 669–689.
