# Supplementary material for: How collective reward structure impedes group decision making: An experimental study using the HoneyComb paradigm
Source: PLoS One. 2021 Nov 16;16(11):e0259963. doi: 10.1371/journal.pone.0259963 (PMC8594797; doi:10.1371/journal.pone.0259963)
Supplement: S4 Text — (PDF) [file pone.0259963.s007.pdf]

Auguie, B. (2017). *gridExtra: Miscellaneous Functions for "Grid" Graphics*. https://CRAN.R-project.org/package=gridExtra

Bates, D., Mächler, M., Bolker, B., & Walker, S. (2015). Fitting Linear Mixed-Effects Models Using lme4. *Journal of Statistical Software*, *67*(1), 1–48. https://doi.org/10.18637/jss.v067.i01

Csardi, G., & Nepusz, T. (2006). The igraph software package for complex network research. *InterJournal*, *Complex Systems*, 1695.

Egeler, P. (2017). *SamplesizeCMH: Power and Sample Size Calculation for the Cochran-Mantel-Haenszel Test*. https://CRAN.R-project.org/package=samplesizeCMH

Epskamp, S., Cramer, A. O. J., Waldorp, L. J., Schmittmann, V. D., & Borsboom, D. (2012). qgraph: Network Visualizations of Relationships in Psychometric Data. *Journal of Statistical Software*, *48*(4), 1–18.

Fox, J., & Weisberg, S. (2019). *An R Companion to Applied Regression* (Third). Sage. https://socialsciences.mcmaster.ca/jfox/Books/Companion/

Green, P., & MacLeod, C. J. (2016). SIMR: An R package for power analysis of generalized linear mixed models by simulation. *Methods in Ecology and Evolution*, *7*(4), 493–498. https://doi.org/10.1111/2041-210X.12504

Henry, L., & Wickham, H. (2020). *purrr: Functional Programming Tools*. https://CRAN.R-project.org/package=purrr

Hervé, M. (2021). *RVAideMemoire: Testing and Plotting Procedures for Biostatistics*. https://CRAN.R-project.org/package=RVAideMemoire

Kuznetsova, A., Brockhoff, P. B., & Christensen, R. H. B. (2017). lmerTest Package: Tests in Linear Mixed Effects Models. *Journal of Statistical Software*, *82*(13), 1–26. https://doi.org/10.18637/jss.v082.i13

Lüdecke, D. (2020). *sjPlot: Data Visualization for Statistics in Social Science*. https://CRAN.R-project.org/package=sjPlot

Makowski, D., Ben-Shachar, M. S., Patil, I., & Lüdecke, D. (2020). Automated reporting as a practical tool to improve reproducibility and methodological best practices adoption. *CRAN*. https://github.com/easystats/report

Neuwirth, E. (2014). *RColorBrewer: ColorBrewer Palettes*. https://CRAN.R-project.org/package=RColorBrewer

R Core Team. (2020). *R: A Language and Environment for Statistical Computing*. R Foundation for Statistical Computing. https://www.R-project.org/

Rosario, H. D. (2021). *Heliosdrm/pwr* [R]. https://github.com/heliosdrm/pwr (Original work published 2015)

RStudio Team. (2020). *RStudio: Integrated Development Environment for R*. RStudio, PBC. http://www.rstudio.com/

Signorell, A., & others, mult. (2020). *DescTools: Tools for Descriptive Statistics*. https://cran.r-project.org/package=DescTools

Tingley, D., Yamamoto, T., Hirose, K., Keele, L., & Imai, K. (2014). mediation: R Package for Causal Mediation Analysis. *Journal of Statistical Software*, *59*(5), 1–38.

Wickham, H. (2011). The Split-Apply-Combine Strategy for Data Analysis. *Journal of Statistical Software*, *40*(1), 1–29.

Wickham, H. (2016). *ggplot2: Elegant Graphics for Data Analysis*. Springer-Verlag New York. https://ggplot2.tidyverse.org

Wickham, H., François, R., Henry, L., & Müller, K. (2020). *dplyr: A Grammar of Data Manipulation*. https://CRAN.R-project.org/package=dplyr

Wilke, C. O. (2020). *cowplot: Streamlined Plot Theme and Plot Annotations for "ggplot2."* https://CRAN.R-project.org/package=cowplot

Xie, Y. (2013). animation: An R Package for Creating Animations and Demonstrating Statistical Methods. *Journal of Statistical Software*, *53*(1), 1–27.
