# Supplementary material for: How collective reward structure impedes group decision making: An experimental study using the HoneyComb paradigm
Source: PLoS One. 2021 Nov 16;16(11):e0259963. doi: 10.1371/journal.pone.0259963 (PMC8594797; doi:10.1371/journal.pone.0259963)
Supplement: S5 Text — (PDF) [file pone.0259963.s008.pdf]

S7. Description of how networks and transitivity were computed.

The network is defined by the nodes (participants) and the edges between the nodes (closeness at the end of one round). With the networks, we aim to represent the closeness on the virtual playing field between two given players and how this changes throughout the game.

1. **Calculate Weights Matrix after each round**

   In order to compute the closeness between two players, the coordinated of each player at the end of a round are extracted. Then the shortest path between these two players is calculated according to P-/ ∞-Norm of moves on a chess board:

   Path from *(0,0)* to *(i,j)*
   where (I,j) are the end coordinates of a given player in a given round:
   i and *j* same sign: *path = |i|+|j|*
   i and *j* diffent sign: *path = max(|i|,|j|)*

   The weight of an edge between two players is then the inverse of the length of the shortest path + 1.
   Example 1: The shortest path between Participant A and B is 3 fields. The closeness of A and B, recorded as the weight of the edge between node A and B, is then 1 / (3+1) = 0.25.

   Example 2: The shortest path between Participant A and C is 0 fields, so both players arrived on the same field. The closeness of A and C, recorded as the weight of the edge between node A and B, is then 1 / (0+1) = 1.

2. **Aggregate weights matrices**

   Because the weights matrices can drastically change between to rounds following each other, they were aggregated as a moving average of the last 5 rounds in order to reflect the dynamic change within the group.
   This means that the resulting weights matrix of round 5 is the average of round 1 through 5, the weights matrix of round 6 is the average of round 2 through 6 and so on. Accordingly, rounds 1 through 4 are not represented in the analysis of closeness between participants.

3. **Compute Global Clustering Coefficient (Transitivity)**

   In order to calculate the global clustering coefficient (transitivity) an unweighted graph is needed. If the weighted networks were directly transformed into unweighted graphs, all participants would have edges to all other participants, thereby rendering the analysis of the network obsolete. Therefore, all edges with weights below the median weight were removed from the network. The resulting sparser network was then transformed into an

unweighted graph that could be used to calculate the global clustering coefficient (transitivity).
