# Supplementary material for: How collective reward structure impedes group decision making: An experimental study using the HoneyComb paradigm
Source: PLoS One. 2021 Nov 16;16(11):e0259963. doi: 10.1371/journal.pone.0259963 (PMC8594797; doi:10.1371/journal.pone.0259963)
Supplement: S6 Text — (PDF) [file pone.0259963.s009.pdf]

S9 Text. Description of mediation analysis

For each of the leaders the same procedure was applied: We fitted three models: the total effect (c), the effect of explanatory variables and the dependent variable, without the mediator (b), and the effect of the independent variables on the mediator (a). We describe here the procedure for the competent leader as an example.

A: Mediator model

We fitted a linear mixed model (estimated using REML and nloptwrap optimizer) to predict transitivity with condition and round (formula: transitivity ~ condition * round). The model included round and group as random effects. We did not include participant id as a random effect as transitivity is calculated at the group level and would therefore lead to singularities in the model. The model's total explanatory power is substantial (conditional R^2 = 0.30) and the part related to the fixed effects alone (marginal R^2) is of 0.08. The model's intercept, corresponding to condition = Cohesion and round = 0, is at 0.70 (95% CI [0.64, 0.77], t(2800) = 21.62, p < .001). Within this model:

- The effect of condition [Independent] is statistically non-significant and negative (beta = -2.85e-12, 95% CI [-0.09, 0.09], t(2800) = -6.55e-11, p > .999; Std. beta = 8.63e-14, 95% CI [-0.42, 0.42])

- The effect of round is statistically significant and positive (beta = 3.03e-03, 95% CI [3.87e-04, 5.68e-03], t(2800) = 2.25, p = 0.025; Std. beta = 0.25, 95% CI [-0.11, 0.60])

- The interaction effect of round on condition [Independent] is statistically non-significant and positive (beta = 1.19e-13, 95% CI [-3.55e-03, 3.55e-03], t(2800) = 6.57e-11, p > .999; Std. beta = -9.85e-14, 95% CI [-0.48, 0.48])

B: Model without Mediator

We fitted a logistic mixed model (estimated using ML and BOBYQA optimizer) to predict Following the competent leader with condition and round (formula: Following the competent leader (0 or 1) ~ condition * round). The model included round and group as random effects. We did not enter participant id as a random effect as the mediation model could not include this effect and the models need to be nested. The model's total explanatory power is substantial (conditional R^2 = 0.54) and the part related to the fixed effects alone (marginal R^2) is of 0.27. The model's intercept, corresponding to condition = Cohesion and round = 0, is at -1.94 (95% CI [-3.13, -0.76], p = 0.001). Within this model:

- The effect of condition [Independent] is statistically non-significant and positive (beta = 1.12, 95% CI [-0.41, 2.65], p = 0.152; Std. beta = 2.74, 95% CI [1.49, 3.98])

- The effect of round is statistically non-significant and negative (beta = -0.05, 95% CI [-0.10, 0.01], p = 0.110; Std. beta = -0.34, 95% CI [-0.77, 0.08])

- The interaction effect of round on condition [Independent] is statistically significant and positive (beta = 0.09, 95% CI [0.02, 0.16], p = 0.010; Std. beta = 0.69, 95% CI [0.16, 1.23])

C: Total model

We fitted a logistic mixed model (estimated using ML and BOBYQA optimizer) to predict Following the competent leader with condition, round and transitivity (formula: Following the competent leader (0 or 1) ~ condition * round * transitivity). The model included round and group as random effects. As in Model B, we could not include the participant id as a random effect as the transitivity model could not include it either. The model's total explanatory power is substantial (conditional R^2 = 0.55) and the part related to the fixed effects alone (marginal R^2) is of 0.28. The model's intercept, corresponding to condition = Cohesion, round = 0 and transitivity = 0, is at -6.33 (95% CI [-10.74, -1.92], p = 0.005). Within this model:

- The effect of condition [Independent] is statistically significant and positive (beta = 6.24, 95% CI [1.02, 11.46], p = 0.019; Std. beta = 2.71, 95% CI [1.45, 3.97])

- The effect of round is statistically non-significant and positive (beta = 0.15, 95% CI [-0.11, 0.40], p = 0.263; Std. beta = -0.44, 95% CI [-0.88, -1.45e-03])

- The effect of transitivity is statistically significant and positive (beta = 6.13, 95% CI [0.31, 11.95], p = 0.039; Std. beta = 0.13, 95% CI [-0.14, 0.39])

- The interaction effect of round on condition [Independent] is statistically non-significant and negative (beta = -0.17, 95% CI [-0.47, 0.12], p = 0.254; Std. beta = 0.79, 95% CI [0.24, 1.34])

- The interaction effect of transitivity on condition [Independent] is statistically significant and negative (beta = -7.12, 95% CI [-13.99, -0.25], p = 0.042; Std. beta = -0.06, 95% CI [-0.37, 0.25])

- The interaction effect of transitivity on round is statistically non-significant and negative (beta = -0.27, 95% CI [-0.60, 0.06], p = 0.111; Std. beta = -0.19, 95% CI [-0.42, 0.04])

- The interaction effect of transitivity on (condition [Independent] * round) is statistically non-significant and positive (beta = 0.37, 95% CI [-0.02, 0.76], p = 0.062; Std. beta = 0.26, 95% CI [-0.01, 0.52])

Standardized parameters were obtained by fitting the model on a standardized version of the dataset. 95% Confidence Intervals (CIs) and p-values were computed using the Wald approximation.

The (partial) mediation effects was then computed using the R package mediation (Tingley et al., 2014).

We checked for mediation of the effect condition: There was a significant total effect (b = 0.378, 95% CI [0.206, 0.53], p < .001), a significant direct effect (b = 0.378, 95% CI [0.206, 0.53], p < .001), but no mediation effect (b < .001, 95% CI [<.001, -0.01], p = 0.98).

We checked for mediation of the effect round: There was no significant total effect (b < -0.001, 95% CI [-0.006, 0.01], p = .94), no significant direct effect (b > -0.001, 95% CI [> -0.001, 0.01], p = .97), and no mediation effect (b > -.001, 95% CI [-.002, -0.00], p = 0.91).

We repeat this analysis for the dependent variables of following the secure, risky, and incompetent leader and receive similar results.
